# Supplementary material for: A Likelihood Approach for Real-Time Calibration of Stochastic Compartmental Epidemic Models
Source: PLoS Comput Biol. 2017 Jan 17;13(1):e1005257. doi: 10.1371/journal.pcbi.1005257 (PMC5240920; doi:10.1371/journal.pcbi.1005257)
Supplement: S1 File — (TAR.GZ) [file pcbi.1005257.s014.tar.gz › HSPH_Online-SI-Revision/Additions/IPoi10/n100-extreme-SEITR/n100-extreme-SEITR_BENCH_table-4.pdf]

1	R0	{1.38654, 9.85565}	{2.64334, 17.2462}	{{2.64334, 17.2462}, {2.64334, 17.2462}}	{90.6429, 74.988}	inf	4942.	15 613.3	{2886., 24 957.}	124.76	0.393235	newly	7106.	7065.83	{1544., 10 240.}	36.2858	0.235651	Reff	1.09645	1.82643	{1.18826, 2.51812}	67.4	ar	92 379.	90 977.	{90 775., 91 176.}	1.51766
2	R0	{2.36629, 12.2029}	{2.7943, 13.5962}	{{2.7943, 13.5962}, {2.7943, 13.5962}}	{18.0878, 11.4178}	inf	1894.	888.841	{50., 2169.}	71.3196	0.829234	newly	3034.	727.524	{46., 1749.}	76.7911	0.932469	Reff	2.22479	2.7552	{2.69969, 2.79192}	23.8412	ar	99 022.	92 455.1	{92 277., 92 642.}	6.63172
3	R0	{2.32253, 12.836}	{2.92239, 11.5217}	{{2.85038, 10.7113}, {2.93597, 11.6547}}	{25.8277, 10.2394}	inf	1753.	1259.96	{110., 2935.}	58.9778	0.615954	newly	2684.	1381.89	{112., 3169.}	57.4241	0.586282	Reff	2.19238	2.87055	{2.78149, 2.93016}	30.9335	ar	98 997.	93 528.1	{93 082., 93 734.}	5.5243
4	R0	{1.79075, 11.2058}	{2.6266, 11.0527}	{{2.6266, 11.0527}, {2.6266, 11.0527}}	{46.676, 1.36626}	inf	2803.	4800.52	{687., 9137.}	73.5775	0.357628	newly	3933.	4486.97	{769., 8222.}	65.9831	0.330762	Reff	1.61667	2.41312	{2.21559, 2.59605}	49.265	ar	96 920.	90 783.9	{90 583., 90 985.}	6.33111
5	R0	{2.26952, 9.62371}	{2.00629, 6.32203}	{{1.90341, 5.70177}, {2.65613, 9.8939}}	{15.8349, 35.9062}	inf	1270.	510.02	{89., 1185.}	74.1631	0.742276	newly	3231.	1064.88	{169., 2528.}	67.4625	0.654706	Reff	2.17415	1.99215	{1.85514, 2.66364}	14.2143	ar	98 881.	79 002.9	{76 533., 91 316.}	20.1031
6	R0	{1.57191, 6.89004}	{2.52921, 10.0481}	{{2.37615, 9.11994}, {2.9884, 12.8326}}	{60.9006, 45.8352}	inf	1343.	944.088	{200., 2018.}	60.0902	0.5105	newly	3690.	1115.3	{229., 2294.}	70.0014	0.681945	Reff	1.49482	2.53982	{2.30565, 2.96333}	69.908	ar	95 046.	89 691.7	{87 333., 94 060.}	5.63338
7	R0	{1.99226, 17.2339}	{2.64334, 17.2462}	{{2.64334, 17.2462}, {2.64334, 17.2462}}	{32.6805, 0.071371}	inf	7484.	13 621.8	{2933., 24 471.}	51.9861	0.287264	newly	6848.	6257.	{1573., 10 142.}	40.6044	0.248476	Reff	1.39466	1.94525	{1.24279, 2.51736}	43.3065	ar	97 992.	90 947.5	{90 762., 91 153.}	7.18882
8	R0	{2.63369, 15.1879}	{2.87181, 13.004}	{{2.87181, 13.004}, {2.87181, 13.004}}	{9.04131, 14.3792}	inf	3695.	1735.7	{150., 5031.}	72.9439	0.843165	newly	4729.	1498.88	{138., 4211.}	70.8957	0.788781	Reff	2.31741	2.7943	{2.65103, 2.86512}	20.5788	ar	99 451.	91 720.9	{92 956., 93 289.}	7.77279
9	R0	{2.53271, 9.90552}	{2.68374, 11.2101}	{{2.6266, 11.0527}, {2.80569, 11.6897}}	{6.87844, 14.1244}	inf	823.	343.662	{31., 812.}	72.7685	0.797715	newly	2227.	375.323	{25., 904.}	83.1467	1.0111	Reff	2.46668	2.66089	{2.59553, 2.80092}	8.70811	ar	99 355.	91 168.7	{90 564., 92 560.}	8.23945
10	R0	{2.48791, 18.9003}	{2.99911, 15.6207}	{{2.99911, 15.6207}, {2.99911, 15.6207}}	{20.5474, 17.3521}	inf	5017.	13 752.4	{3174., 24 984.}	93.6873	0.368238	newly	4646.	7567.73	{2084., 12 541.}	88.2495	0.346033	Reff	2.05964	2.29444	{1.6523, 2.85428}	22.3016	ar	99 291.	94 050.8	{93 925., 94 166.}	5.27759
11	R0	{2.84414, 18.4765}	{2.98125, 18.4749}	{{2.98125, 18.4749}, {2.98125, 18.4749}}	{4.82079, 0.00865965}	inf	3971.	4897.49	{635., 10 685.}	57.1815	0.440792	newly	4035.	2500.13	{352., 5270.}	53.7514	0.464851	Reff	2.47881	2.75339	{2.47921, 2.95284}	12.3816	ar	99 654.	93 928.5	{93 796., 94 043.}	5.74535
12	R0	{2.29369, 14.622}	{2.86605, 14.6757}	{{2.86193, 14.6465}, {2.86193, 14.6465}}	{24.9535, 0.367433}	inf	4819.	3696.57	{571., 8059.}	58.5418	0.513465	newly	5911.	2584.81	{416., 5447.}	58.8604	0.519179	Reff	1.91186	2.69858	{2.48919, 2.8374}	41.1496	ar	98 982.	93 049.	{92 830., 93 190.}	5.99406
13	R0	{2.73413, 16.7529}	{2.7943, 13.5962}	{{2.7943, 13.5962}, {2.7943, 13.5962}}	{2.2007, 18.8427}	inf	4263.	4143.03	{521., 9050.}	58.3667	0.53136	newly	4789.	3096.26	{412., 6557.}	55.4802	0.490646	Reff	2.35852	2.60708	{2.38426, 2.77231}	11.6569	ar	99 543.	92 445.2	{92 280., 92 597.}	7.13041
14	R0	{1.79554, 16.5943}	{2.41197, 16.4137}	{{2.41197, 16.4137}, {2.41197, 16.4137}}	{34.3312, 1.08833}	inf	6015.	13 864.1	{3079., 21 303.}	71.8711	0.363787	newly	5421.	6482.3	{1753., 9386.}	53.7556	0.315476	Reff	1.3756	1.69069	{1.18761, 2.28546}	29.0871	ar	96 821.	88 031.7	{87 750., 88 338.}	9.07793
15	R0	{1.91429, 13.7771}	{2.7811, 19.6712}	{{2.7811, 19.6712}, {2.7811, 19.6712}}	{45.281, 42.7819}	inf	5411.	11 627.8	{3022., 21 109.}	69.859	0.305118	newly	6031.	4890.35	{1505., 8396.}	39.9602	0.281442	Reff	1.53354	2.21571	{1.71588, 2.64238}	45.7131	ar	97 541.	92 342.9	{92 182., 92 485.}	5.32915
16	R0	{1.42094, 10.5871}	{2.58655, 14.3523}	{{2.58655, 14.3523}, {2.58655, 14.3523}}	{82.0309, 35.564}	inf	3733.	13 593.1	{3107., 22 545.}	159.681	0.411919	newly	4920.	7693.25	{2212., 11 616.}	79.6707	0.289676	Reff	1.20628	1.90017	{1.36645, 2.45523}	58.9889	ar	93 044.	90 309.9	{90 076., 90 481.}	2.93849
17	R0	{1.45515, 6.16466}	{2.72175, 10.8305}	{{2.68409, 10.6354}, {2.91946, 11.8546}}	{87.0425, 75.6864}	inf	733.	344.746	{39., 785.}	67.7319	0.676868	newly	2188.	403.984	{46., 841.}	81.5364	0.96636	Reff	1.41733	2.69907	{2.65677, 2.88577}	90.4334	ar	93 254.	91 655.8	{91 166., 93 476.}	1.79578
18	R0	{2.85669, 13.2416}	{2.67672, 12.1364}	{{2.52844, 11.113}, {2.95209, 14.037}}	{8.63771, 12.5512}	inf	1540.	540.619	{39., 1106.}	80.944	0.998656	newly	2807.	508.27	{36., 1105.}	82.5713	1.06632	Reff	2.71777	2.65988	{2.48844, 2.94722}	7.6638	ar	99 644.	89 648.9	{89 331., 93 751.}	10.0309
19	R0	{2.56417, 17.9558}	{2.97157, 19.3547}	{{2.97157, 19.3547}, {2.97157, 19.3547}}	{15.8882, 7.7908}	inf	4153.	3240.15	{233., 7239.}	59.6371	0.590113	newly	4054.	1600.04	{117., 3518.}	63.1909	0.650227	Reff	2.21442	2.82276	{2.63525, 2.9606}	27.4718	ar	99 389.	93 841.9	{93 684., 93 992.}	5.58121
20	R0	{2.27517, 11.1864}	{2.81598, 11.7741}	{{2.80569, 11.6897}, {2.9884, 12.8326}}	{23.7701, 5.45283}	inf	2051.	729.766	{116., 1752.}	76.2601	0.848818	newly	3692.	764.156	{133., 1816.}	79.3024	0.904388	Reff	2.12451	2.77831	{2.70233, 2.80547}	30.7743	ar	98 894.	91 107.1	{92 237., 92 862.}	7.87399
21	R0	{2.35735, 10.4735}	{2.75567, 11.0134}	{{2.68409, 10.6354}, {2.91946, 11.8546}}	{16.897, 5.15448}	inf	1087.	442.855	{55., 1118.}	74.7068	0.845372	newly	2417.	502.968	{59., 1181.}	79.1904	0.965143	Reff	2.2751	2.74172	{2.6489, 2.91307}	20.5097	ar	99 036.	92 066.2	{91 232., 93 550.}	7.03762
22	R0	{1.79551, 11.9179}	{2.81617, 13.9425}	{{2.81617, 13.9425}, {2.81617, 13.9425}}	{56.8451, 16.9879}	inf	4439.	13 773.9	{3445., 24 686.}	122.982	0.360545	newly	5958.	8343.27	{2657., 13 354.}	70.1071	0.274491	Reff	1.5037	2.11636	{1.4681, 2.65928}	43.3018	ar	97 033.	92 605.7	{92 459., 92 769.}	4.56267
23	R0	{1.23545, 6.31687}	{2.7628, 12.2542}	{{2.47628, 10.2848}, {2.87181, 13.004}}	{123.627, 93.9919}	inf	2814.	4889.29	{597., 10 426.}	76.5401	0.393274	newly	6598.	4140.17	{538., 8232.}	50.0255	0.438468	Reff	1.0892	2.56001	{2.25279, 2.84272}	135.037	ar	88 739.	92 137.8	{88 885., 93 208.}	3.84426
24	R0	{2.24089, 10.1789}	{2.7943, 13.5962}	{{2.7943, 13.5962}, {2.7943, 13.5962}}	{24.696, 33.5724}	inf	976.	419.217	{37., 1104.}	73.1578	0.830753	newly	2027.	345.58	{33., 922.}	82.9512	1.06799	Reff	2.17337	2.77612	{2.74627, 2.79276}	27.7334	ar	98 868.	92 452.6	{92 318., 92 588.}	6.48889
25	R0	{1.57298, 10.2607}	{2.58655, 14.3523}	{{2.58655, 14.3523}, {2.58655, 14.3523}}	{64.4363, 39.8764}	inf	2641.	3263.16	{611., 7011.}	59.1809	0.421049	newly	3941.	2219.	{442., 4604.}	55.1604	0.493335	Reff	1.41523	2.44503	{2.28659, 2.56024}	72.7661	ar	94 957.	90 326.7	{90 158., 90 528.}	4.87621
26	R0	{1.2154, 8.2576}	{2.8833, 17.1847}	{{2.8833, 17.1847}, {2.8833, 17.1847}}	{137.231, 108.108}	inf	4010.	7875.06	{1767., 13 655.}	73.2997	0.310592	newly	6408.	4184.4	{1065., 7062.}	42.9566	0.329079	Reff	0.993966	2.51307	{2.23355, 2.80363}	152.832	ar	88 421.	93 188.8	{93 040., 93 339.}	5.39211
27	R0	{2.22841, 18.7983}	{2.52651, 14.1724}	{{2.52651, 14.1724}, {2.52651, 14.1724}}	{13.3773, 24.6081}	inf	7582.	14 907.3	{3434., 23 271.}	51.7865	0.31729	newly	6511.	8118.53	{2494., 11 507.}	56.352	0.294844	Reff	1.58429	1.716	{1.05211, 2.37681}	27.2614	ar	98 756.	89 555.3	{89 350., 89 769.}	9.31661
28	R0	{1.27778, 5.36767}	{2.46369, 8.47708}	{{2.21381, 7.10188}, {2.74924, 10.0386}}	{92.8103, 57.9284}	inf	966.	2294.6	{279., 5184.}	121.503	0.416473	newly	2971.	3437.14	{369., 7237.}	66.9355	0.36387	Reff	1.23118	2.3599	{2.0507, 2.73745}	91.6783	ar	89 843.	88 013.4	{84 375., 92 227.}	4.30214
29	R0	{2.38042, 15.8901}	{2.81617, 13.9425}	{{2.81617, 13.9425}, {2.81617, 13.9425}}	{18.3056, 12.2567}	inf	3337.	8787.63	{1979., 16 779.}	101.914	0.344193	newly	3642.	5868.15	{1668., 10 530.}	91.3667	0.315983	Reff	2.11469	2.40172	{1.99869, 2.72954}	16.894	ar	99 112.	92 668.	{92 498., 92 819.}	6.50177
30	R0	{2.53188, 12.7607}	{2.80098, 11.6736}	{{2.80569, 11.6897}, {2.80569, 11.6897}}	{10.6284, 8.5307}	inf	1016.	376.544	{57., 800.}	73.3137	0.762371	newly	1649.	400.702	{59., 814.}	75.7003	0.799609	Reff	2.45506	2.78693	{2.77191, 2.80328}	13.5175	ar	99 317.	92 492.3	{92 374., 92 678.}	6.87164
31	R0	{1.24482, 10.5471}	{2.65223, 17.3283}	{{2.64334, 17.2462}, {2.64334, 17.2462}}	{113.062, 64.2941}	inf	5483.	17 363.1	{5365., 25 322.}	118.606	0.374894	newly	6713.	7622.84	{2967., 10 168.}	39.3491	0.217245	Reff	0.914831	1.68712	{1.00566, 2.41242}	85.0277	ar	89 097.	91 040.6	{90 829., 91 192.}	2.18146
32	R0	{2.30446, 17.9607}	{2.7943, 13.5962}	{{2.7943, 13.5962}, {2.7943, 13.5962}}	{21.2562, 24.3003}	inf	5816.	9278.73	{1557., 19 685.}	58.1013	0.358376	newly	5244.	6220.24	{1243., 11 999.}	62.57	0.307818	Reff	1.82382	2.34909	{1.8071, 2.72556}	31.1327	ar	99 028.	92 449.8	{92 296., 92 619.}	6.64276
33	R0	{2.31579, 11.8236}	{2.77472, 9.36216}	{{2.37035, 7.13655}, {2.9194, 10.1535}}	{19.8173, 20.8181}	inf	3092.	10 609.5	{2338., 21 131.}	146.962	0.40133	newly	5187.	11 131.8	{3036., 19 019.}	130.82	0.374329	Reff	2.08296	2.26974	{1.64237, 2.78055}	19.4931	ar	99 012.	92 040.	{87 398., 93 604.}	7.04154
34	R0	{1.6678, 10.6744}	{2.7943, 13.5962}	{{2.7943, 13.5962}, {2.7943, 13.5962}}	{67.5441, 27.372}	inf	4713.	9822.1	{1639., 18 924.}	80.7852	0.346081	newly	6918.	6570.08	{1308., 11 756.}	48.2583	0.297065	Reff	1.37288	2.32583	{1.85103, 2.72196}	69.4121	ar	95 809.	92 434.5	{92 238., 92 608.}	3.52212
35	R0	{1.65476, 7.07559}	{2.69821, 10.7086}	{{2.68409, 10.6354}, {2.68409, 10.6354}}	{63.0576, 51.345}	inf	893.	425.627	{42., 1179.}	71.0596	0.764831	newly	2514.	495.322	{52., 1354.}	80.5442	0.988475	Reff	1.60409	2.68606	{2.63449, 2.68398}	67.4506	ar	95 746.	90 029.8	{91 143., 91 794.}	5.97017
36	R0	{2.71807, 18.5959}	{2.58655, 14.3523}	{{2.58655, 14.3523}, {2.58655, 14.3523}}	{4.83873, 22.8201}	inf	3397.	1453.97	{129., 2828.}	74.6248	0.795614	newly	3163.	1033.41	{88., 2069.}	70.4814	0.715167	Reff	2.4286	2.52415	{2.4641, 2.58047}	4.31948	ar	99 540.	90 297.1	{90 071., 90 530.}	9.28558
37	R0	{2.6202, 12.7552}	{2.5065, 14.0053}	{{2.48103, 13.7658}, {2.48103, 13.7658}}	{5.71747, 9.80087}	inf	1248.	751.3	{24., 1469.}	65.9338	0.722968	newly	2178.	555.4	{13., 1070.}	75.8724	0.925085	Reff	2.51914	2.48369	{2.41315, 2.48048}	3.80006	ar	99 426.	89 327.1	{88 720., 89 408.}	10.1572
38	R0	{1.47672, 9.81651}	{2.58655, 14.3523}	{{2.58655, 14.3523}, {2.58655, 14.3523}}	{75.1551, 46.2057}	inf	4210.	9520.27	{2558., 18 499.}	82.6413	0.315921	newly	6266.	5791.32	{1834., 10 343.}	43.023	0.259949	Reff	1.23	2.14183	{1.68615, 2.47704}	74.1322	ar	93 815.	90 382.9	{90 106., 90 570.}	3.65842
39	R0	{1.90004, 12.3313}	{2.99911, 15.6207}	{{2.99911, 15.6207}, {2.99911, 15.6207}}	{57.8446, 26.6752}	inf	3615.	6631.24	{797., 14 571.}	71.9659	0.370536	newly	4980.	4108.34	{517., 8427.}	51.7998	0.374543	Reff	1.65284	2.68412	{2.28826, 2.95685}	62.3941	ar	97 413.	94 030.	{93 893., 94 162.}	3.47286
40	R0	{2.64218, 18.5237}	{2.86895, 12.986}	{{2.85273, 12.8842}, {2.87181, 13.004}}	{8.58261, 29.8951}	inf	6412.	14 521.5	{4126., 25 098.}	66.2067	0.283006	newly	6088.	9624.27	{3530., 14 635.}	77.6885	0.279226	Reff	2.0572	2.13027	{1.52424, 2.68256}	18.986	ar	99 474.	93 083.5	{92 928., 93 221.}	6.42434
41	R0	{1.6945, 7.17996}	{2.85381, 13.4925}	{{2.86001, 13.5542}, {2.86001, 13.5542}}	{68.4161, 87.9189}	inf	498.	219.971	{17., 518.}	70.6766	0.711937	newly	1333.	190.058	{15., 439.}	85.7421	1.07683	Reff	1.66739	2.84146	{2.83387, 2.85901}	70.414	ar	96 332.	92 898.1	{92 821., 93 173.}	3.56468
42	R0	{1.84559, 18.0074}	{2.64334, 17.2462}	{{2.64334, 17.2462}, {2.64334, 17.2462}}	{43.2247, 4.22715}	inf	7319.	13 815.9	{5209., 22 716.}	41.7524	0.213306	newly	6151.	6464.17	{2805., 9937.}	36.2966	0.185767	Reff	1.29117	1.95712	{1.43216, 2.41432}	52.7924	ar	97 358.	90 969.9	{90 711., 91 184.}	6.56147
43	R0	{2.24965, 17.0361}	{2.61082, 12.9924}	{{2.44675, 11.6528}, {2.7346, 14.003}}	{16.0547, 23.736}	inf	4414.	12 164.5	{1645., 22 499.}	104.198	0.398774	newly	4281.	7882.	{1284., 12 621.}	113.319	0.394687	Reff	1.90728	2.03315	{1.48243, 2.62092}	18.975	ar	98 770.	90 705.3	{88 380., 92 051.}	8.16512
44	R0	{2.54324, 15.7967}	{2.7346, 14.003}	{{2.7346, 14.003}, {2.7346, 14.003}}	{7.52426, 11.3549}	inf	4842.	3236.2	{238., 8188.}	64.4593	0.660484	newly	5449.	2340.03	{185., 5638.}	62.2231	0.631388	Reff	2.13836	2.59036	{2.3628, 2.72385}	21.3153	ar	99 354.	91 938.6	{91 791., 92 120.}	7.46365
45	R0	{2.54943, 14.8032}	{2.87181, 13.004}	{{2.87181, 13.004}, {2.87181, 13.004}}	{12.6452, 12.1541}	inf	3565.	1509.4	{224., 3456.}	73.2044	0.791507	newly	4552.	1333.66	{203., 3016.}	70.7018	0.744932	Reff	2.25387	2.80511	{2.71814, 2.86199}	24.4571	ar	99 380.	93 099.5	{92 941., 93 239.}	6.3197
46	R0	{2.37755, 12.6461}	{2.93597, 11.6547}	{{2.93597, 11.6547}, {2.93597, 11.6547}}	{23.4872, 7.83957}	inf	1629.	4291.86	{483., 9011.}	108.499	0.390627	newly	2717.	4164.57	{545., 8247.}	94.355	0.365978	Reff	2.25026	2.73848	{2.51639, 2.91389}	21.7869	ar	99 096.	93 582.2	{93 407., 93 747.}	5.56408
47	R0	{1.69279, 11.6636}	{2.5684, 13.7324}	{{2.5684, 13.7324}, {2.5684, 13.7324}}	{51.7258, 17.7372}	inf	3683.	7363.47	{561., 17 061.}	87.1338	0.418397	newly	4864.	4777.8	{429., 10 271.}	62.0637	0.391975	Reff	1.4668	2.22664	{1.73655, 2.54261}	52.0977	ar	96 214.	90 118.2	{89 906., 90 318.}	6.33565
48	R0	{1.82402, 10.0038}	{2.93091, 13.8908}	{{2.95209, 14.037}, {2.95209, 14.037}}	{60.684, 38.8552}	inf	1751.	3367.99	{530., 7420.}	76.8449	0.324572	newly	3183.	2587.28	{474., 5435.}	53.0038	0.340738	Reff	1.71321	2.78633	{2.60649, 2.92133}	62.638	ar	97 076.	93 599.8	{93 566., 93 864.}	3.58087
49	R0	{1.6424, 11.1667}	{2.95209, 14.037}	{{2.95209, 14.037}, {2.95209, 14.037}}	{79.7425, 25.7041}	inf	5534.	21 102.1	{8964., 29 368.}	144.482	0.370701	newly	7611.	11 717.6	{6396., 14 559.}	64.3098	0.222371	Reff	1.28365	1.75784	{1.07173, 2.53968}	44.9065	ar	95 546.	93 711.1	{93 570., 93 853.}	1.92039
50	R0	{2.05638, 14.3048}	{2.95209, 14.037}	{{2.95209, 14.037}, {2.95209, 14.037}}	{43.5576, 1.8721}	inf	4454.	7124.37	{1375., 14 240.}	59.884	0.328908	newly	5024.	5008.76	{1133., 9501.}	52.9786	0.297633	Reff	1.73898	2.61551	{2.26352, 2.89237}	50.5325	ar	98 194.	93 710.9	{93 563., 93 861.}	4.56553
